# Relevance of β-Glucan Molecular Properties on Its Suitability as Health Promoting Bread Ingredient

**DOI:** 10.3390/nu14081570

**Published:** 2022-04-09

**Authors:** Marcus Schmidt, Elisabeth Sciurba, Sharline Nikolay, Alexandra Hüsken, Inga Smit

**Affiliations:** Department of Safety and Quality of Cereals, Max Rubner-Institut, Federal Research Institute of Nutrition and Food, 32756 Detmold, Germany; elisabeth.sciurba@mri.bund.de (E.S.); sharline.nikolay@mri.bund.de (S.N.); alexandra.huesken@mri.bund.de (A.H.); inga.smit@mri.bund.de (I.S.)

**Keywords:** barley, beta-glucan, molar mass, molar ratio, bread making, functional food, in vitro digestion, viscosity, bile acid binding

## Abstract

The fate of β-glucan (BG) health promoting properties during food production is crucial, but not predictable yet. Therefore, high molar mass BG (hBG) and control BG (cBG) were extracted from two barley varieties, characterized and added to wheat breads at levels of 3% and 6%. Bread quality criteria, carbohydrate contents and BG content and structural properties were determined. Additionally, breads were subjected to an in vitro digestion. The BG content in the chyme, molar mass, molar ratio, viscosity and bile acid retention were determined. The hBG and the cBG decreased loaf volume and increased crumb hardness with increasing BG content. The reduction in BG content during bread making was similar for hBG and cBG, but the molar mass of cBG decreased to a greater extent. As a result, only 10% of cBG entering in vitro digestion were found in the chyme afterwards, while 40% of the ingested hBG were detected. Molar mass reduction was much more severe for cBG compared to hBG. The use of hBG showed higher viscosity and better bile acid retention, indicating cholesterol lowering properties, compared to similar or higher amounts of cBG. These results provide valuable knowledge on the criteria to select BG-rich raw materials for ideal health promoting properties.

## 1. Introduction

According to the recommendation of the European Food Safety Authority (EFSA), adults should consume at least 25 g (better 30 g) of dietary fiber daily to maintain healthy bowel function [1]. As this amount is only reached by a small part of the population, researchers and food manufacturers are undertaking efforts to produce fiber-enriched foods. One type of dietary fiber, which gained particular interest and popularity is cereal β-glucan (BG) [2]. BG is present in numerous cereals, such as barley (3–11%), oats (3–7%), rice (1–2%) and wheat (<1%) [3]. Depending on variety and climate conditions during growth, BG content can vary substantially, even within the same species of cereals [3]. However, barley and oat generally contain with 18.6–53.7 g/kg (based on dry matter, DM) the highest levels and hence, are the most intensively investigated sources of cereal BG [4]. Within the cereal grain, highest concentration (75%) can be found in the cell walls of the endosperm, followed by the aleurone and sub-aleurone layers with a BG proportion of approximately 25% [5].

Regarding the chemical structure, cereal BG composes a heterogeneous group of non-starch polysaccharides build out of glucose. The glucose units form long linear chains, which are classified by their intramolecular linkages consisting of β-(1-3) and β-(1,4) glycosidic bonds between the monomers [6,7,8]. In the most frequently found internal structure, three or four D-glucose monomers are connected by β-(1,4) linkages, followed by a single β-(1,3) bond. However, up to 14 consecutive β-(1,4) linked monomers are reported [9,10]. Each group of β-(1,4) linked glucose monomers is considered a sub-unit, which is connected to the next one by a β-(1,3) linkage. The sub-units are commonly classified according to their degree of polymerization (DP). Sub-units of three or four glucose monomers form cellotriosyl (DP3) or cellotetraosyl (DP4) sub-oligosaccharides, respectively. For the functionality of cereal BG, the ratio between DP3 and DP4 sub-units, referred to as molar ratio, and the molar mass of the whole molecule are the most relevant structural characteristics. Unfortunately, both parameters can vary substantially depending on variety and environmental conditions during grain development [7].

The particular interest in cereal BG originates from the various health-promoting properties, including lowering of low-density lipoprotein (LDL) cholesterol levels and postprandial glycemic response, as well as an increased feeling of satiety [7,11]. There are also some less intensely studied effects, such as a reduced risk for colon cancer [12]. The EFSA and the American Agency for Food and Drug Administration (FDA) recognized the contribution of BG to the maintenance of normal blood LDL cholesterol levels, if at least 3 g of BG are consumed daily [13,14]. Products containing at least 1 g of oat or barley BG per portion, allowing for a daily consumption of 3 g can be advertised with the health claim: “Oat beta-glucan has been shown to lower/reduce blood cholesterol. Blood cholesterol lowering may reduce the risk of (coronary) heart disease.” [14]. In consequence, the use of cereal BG as health promoting food ingredient appears as a great opportunity.

Structural properties, such as molar mass, molar ratio or solubility of the BG present in food products are currently not considered when issuing the health claim. However, it was shown in a number of studies that these properties have an impact on the health-promoting effects. For instance, a high molar mass is associated with the formation of a highly viscous gel in the intestine, which hinders the absorption of free bile acids from the intestine into the body. As a result, the de novo synthesis of bile acids is stimulated, with cholesterol being used as substrate and thus, reducing the risk for cardiovascular disease [15,16]. Also, a high molar mass and low molar ratio was reported to result in better solubility of BG [17]. However, due to the high complexity of the interactions between structure and function of BG, the relationships have not yet been fully elucidated. Although it is known that the molar mass has an influence on the functionality, further details are still unclear. As a result, there are contradicting reports regarding the efficiency of hydrolyzed or low molar mass BG for cholesterol reduction [16,18]. Furthermore, it is well known that properties such as molar mass and solubility can change significantly during food production and storage [17,19]. Common strategies to minimize BG degradation during bread making include the use of more coarse barley flour, but with the drawback of a reduction in in vitro solubility and health promoting benefits [20,21]. Otherwise, proofing time of the dough could be minimized, which could lead to reduced product quality [20,22]. According to recent findings, a high molar ratio provides increased resistance against hydrolytic enzymes, hence preserving the beneficial molar mass better throughout the food production process [23]. Despite all efforts, there is a distinct lack of investigations on the relation between BG structural properties and the changes to BG structure and functionality throughout food production and consumption. Hence, alterations in BG content, molar mass and molar ratio can neither be fully eliminated, nor predicted. As a result, only BG content is considered for the health claim.

It was the aim of this study to contribute to filling the knowledge gap regarding the fate of BG from the raw material over the food product until the human intestine. Therefore, two structurally divers BG were extracted from barley flours and used for the production of BG fortified model wheat breads, which were subjected to an in vitro digestion assay. Following the development of BG content, molar mass and molar ratio throughout the process allows to draw conclusions regarding the fate of BG, depending on its molecular structure in the raw material. Moreover, determination of dynamic viscosity and bile acid retention capacity of the BG fortified breads compared to the standard wheat bread shows the fate of the BG health promoting properties throughout the process, depending on the structural characteristics. Thus, this study provides valuable knowledge on the selection of appropriate BG containing raw materials towards the production of foods with maximum health promoting properties.

## 2. Materials and Methods

### 2.1. Materials

All experiments were undertaken with two commercial de-hulled barley (*Hordeum vulgare)* flours obtained from grains harvested in 2020. One was purchased from Birlin-Mühle GmbH (Rheinfelden-Degerfelden, Germany), and the other one from Mühle Schlingmann e.K. (Waltrop, Germany), which is advertised with enhanced dietary fiber and beta-glucan contents (beta^®^Gerste). Due to the correlation between BG content and molar mass, the latter one was expected to contain BG of higher molar mass [24]. Barley flours were stored in barrels under cool conditions (<20 °C) and were aerated regularly. Wheat flour type 550 (German classification, mineral content according to the supplier 0.51–0.63% dry matter (DM) determined as total ash, crude protein content 12.4% DM [25]) was purchased from Roland Mills North (Bremen, Germany).

Chemicals were acquired from Roth (DE) in analytical quality, unless stated otherwise.

### 2.2. Extraction of β-Glucan from Barley Flours

Extraction of BG from the two barley flours was based on the method of Temelli [26], with some modifications. In brief, whole barley flour was suspended in distilled water (flour:water = 1:10, *w*/*v*). After adjusting the pH to 7, using sodium carbonate (20% *w*/*v*), the suspension was stirred for 30 min at 50–55 °C. The slurry was then cooled to room temperature (RT) and centrifuged (4400× *g*, 4 °C, 30 min). The pH of the supernatant was adjusted to 4.5, using hydrochloric acid (2 M). After centrifugation (4400× *g*, 4 °C, 30 min), BG was precipitated from the supernatant by adding an equal volume of ethanol (97%). The precipitate was allowed to settle overnight at 4 °C and the supernatant was discarded. After re-suspension in ethanol (97%), filtration (Whatman 597 ½, d = 270 mm, pore size 4.0–7.0 µm) and washing with ethanol, the pellet was air-dried overnight under a fume hood. The dried product was ground to pass a 0.5 mm sieve (Retsch, ZM 200, Haan, Germany) and stored at 4 °C until further use. Each extraction was carried out in three independent replicates, which were combined and homogenized for further analysis. BG extract from the beta^®^Gerste was labelled as high molar mass BG (hBG) and from the other variety as control BG (cBG).

Compositional analysis of the BG extracts was carried out, using the following methods. Dry matter of the extracts was determined according to AACC (American Association of Cereal Chemists) International Method 44-15.02 [27], using the one-stage method. The total ash content was determined according to AACC International Method 08-01.01 [28]. Total starch content was determined according to the Ewers method (ISO 10 520:1997) [29]. Crude protein content was determined according to the International Association for Cereal Chemistry (ICC) standard No. 167 [30] using the Dumas combustion method. The content of total pentosans was determined photometrically according to the method of Hashimoto et al. [31].

### 2.3. Production and Evaluation of β-Glucan Fortified Model Wheat Breads

Preparation of model wheat breads with and without fortification with the extracted BG was based on the AACC International method 1-10.03 [32], with some modifications. For the wheat flour, the amount of water (based on flour weight) required for a dough consistency of 500 Brabender units (BU) (determined using a Brabender Farinograph according to the AACC International method 54-21.02 [33]) was equal to 61% and used for all five recipes. For BG fortified breads, fortified wheat flour was prepared by blending flour with hBG or cBG extracts to final BG contents of 3% or 6%, respectively. Breads made with fortified flours containing 3% and 6% BG from hBG or cBG were labelled as hBG 3 and hBG 6 or cBG 3 and cBG 6, respectively. The standard wheat bread was abbreviated as swb.

For all breads, flour was combined with 61% water, 5% yeast (Uniferm GmbH & Co. KG, Werne, Germany), 1.5% salt, (K + S Minerals and Agriculture GmbH, Kassel, Germany), 1% sugar (Nordzucker AG, Brunswig, Germany) and 1% fat (CSM Deutschland GmbH, Bremen, Germany), all based on flour weight. All ingredients were mixed to a dough using a hand mixer (type 3 mix 7000, Krups, Solingen, Gemany) for 1 min at speed level 1 and 3 min at speed level 6. After the dough was allowed to rest in a proofing cabinet (model G 86W, Manz Backtechnik GmbH, Creglingen-Münster, Germany) for 20 min at 30 °C and 80% relative humidity, it was molded and transferred into a loaf tin (dimensions: bottom outside 80 mm × 80 mm, height 100 mm, top outside 130 mm) for the second proofing (30 min, 30 °C, 80% relative humidity). The dough was baked at 200 °C for 45 min, using a domestic oven (type HI3F5R, Bosch, Gerlingen, Germany).

After cooling to room temperature, the loaf volume of the breads was measured by rapeseed displacement according to AACC International Method 10-05.01 [34]. The bake-loss was determined by weighing the dough and resulting bread before and after baking, respectively and expressed as percentage weight reduction during the baking process. To assess the crumb characteristics, the loaves were cut into slices of 30 mm width. The pore structure was evaluated visually, using the pore table by Dallmann [35].

Texture Profile Analysis (TPA, Texture Analyser TA.XTplus, Stable Micro Systems, Godalming, UK), equipped with an aluminum cylinder (25 mm diameter), was used to characterize the crumb texture. The following parameters were used: TPA, pre-test speed: 1 mm/s, test speed: 0.8 mm/s, retest speed: 0.8 mm/s, deformation: 60% of sample height, waiting time between measurements 10 s, release force: 5 g, measuring data rate: 200 data points per second. Moisture content of the breads was determined using a rapid moisture analyzer (MA30, Satorius, Göttingen, Germany) at a temperature of 120 °C until constant weight was reached. The remaining breads were diced (20 × 20 mm) and frozen at −20 °C until further use.

### 2.4. In Vitro Digestion

The breads with and without the addition of BG were subjected to an in vitro digestion model, following the method described by Versantvoort et al. [36]. In brief, 4.5 g of fresh bread was mixed with 6 mL saliva solution and incubated for 5 min at 37 °C. After addition of 12 mL gastric acid solution and incubation for 2 h at 37 °C under constant inversion (55 rpm), 12 mL of duodenal solution, 6 mL of bile acid solution and 2 mL of bicarbonate solution (1 M) were added simultaneously. The mixture was incubated for another 2 h at 37 °C, while rotating overhead (55 rpm). After the digestion procedure was finished, 500 µL of Carrez I (potassium hexacyanoferrate(II) trihydrate, 15%, *w*/*v*) and 500 µL of Carrez II (zinc(II) acetate, 23%, *w*/*v*) were added. The chyme was obtained by centrifugation for 5 min at 2700× *g* (RT) to remove the solids. The chyme samples obtained from in vitro digestion were labelled as digested standard wheat bread (d-swb), digested cBG 3 and 6 (d-cBG 3 and 6) and digested hBG 3 and 6 (d-hBG 3 and 6). 2.5β-glucan content

The BG content was determined according to the AOAC Method 995.16, ICC Standard Method No. 166 and AACC Method 32-23.01 [37], using the K-BGLU mixed linkage β-glucan assay kit, purchased from Megazyme (Bray, Ireland). To determine the BG content in the hBG and cBG extracts, Method A was used, as described in the manual supplied with the test kit. To account for the high BG content, a 4-fold dilution was used after incubation with lichenase. In the wheat breads, BG content was determined following method B of the manual. After in vitro digestion, BG content of the chyme samples was measured following the instructions of method C.

### 2.5. Determination of β-Glucan Molar Mass and Polydispersity

Prior to sample preparation, all solid samples were ground to pass a 0.5 mm sieve (Retsch, ZM 200, Haan, Germany) and homogenized. For the bread samples, approximately 200 mg were mixed with 5 mL of aqueous ethanol (50% *v*/*v*) and placed for 5 min in a boiling water bath, before another 5 mL of the ethanol (50% *v*/*v*) were added. After centrifugation (10 min, 1800× *g*, RT) the supernatant was discarded and the pellet washed with 10 mL of ethanol (50% *v*/*v*).

For the chyme, after in vitro digestion, 3 mL were boiled for 5 min and allowed to cool to room temperature. After addition of 3 mL aqueous ethanol (95% *v*/*v*), the mixture was homogenized and another 5 mL of the ethanol solution were added. The pellet was recovered by centrifugation (10 min, 1800× *g*, RT) and washed with 8 mL of aqueous ethanol (50% *v*/*v*).

The so prepared bread and chyme samples, as well as portions of the original BG extracts (hBG and cBG) were suspended in sodium nitrate solution (0.1 M) containing 0.02% (*w*/*v*) sodium azide to a final BG concentration of approximately 1 mg/mL. The suspension was stirred at 90 °C for 2.5 h. After cooling, 250 µL of α-amylase solution (~5 mg/mL dissolved in 3.6 mM CaCl_2_ solution) were added and incubated in a water bath (37 °C) for 1 h to ensure absence of starch in the samples [19]. To remove proteins, 50 µL Carrez I (15%, *w*/*v*) and 50 µL Carrez II (23%, *w*/*v*) were added. The supernatants were filtered through a pre-syringe filter (0.45 µm) into a gel permeation chromatography (GPC) vial. BG molar mass standards (M_w_ = 650 kDa, 392 kDa, 265 kDa, 229 kDa, 70.6 kDa, 35.6 kDa) were obtained from Megazyme (Bray, Ireland) and prepared similar to the hBG and cBG extracts.

Determination of the molar mass and dispersity was carried out using the GPCmax gel permeation chromatography system (Malvern Panalytical, Enigma, UK). The system was equipped with a Viscotek TDA 305 multidetector (Malvern Panalytical, Enigma, UK) with refractive index (RI) detector. Separation was carried out on A6000M (300 × 8 mm), Aq GPC/SEC double column, equipped with an AGuard (50 × 6 mm) pre-column. The measurement was carried out isocratic using 0.1 M sodium nitrate solution, containing 0.02% sodium azide, at a flow rate of 1 mL/min. The column temperature was held at 30 °C and the run time was 35 min. Injection volume of the sample was 100 µL. For data evaluation and conventional calibration, based on the RI-signal of the discreet BG molecular weight standards, OmniSEC 5.10 software from Malvern was used.

Dispersity and average molar masses were calculated according to the following formulas, using OmniSEC 5.10 software.

(1) $$M_{n}=\frac{\sum_{i=1}^{n}N_{i}\times {\;M}_{i}}{N}$$

(2) $$M_{w}=\frac{\sum_{i=1}^{n}m_{i}\times {\;M}_{i}}{m}$$

(3) $$\mathrm{dispersity}=\frac{M_{w}}{M_{n}}$$

Thereby, M_n_ = number average molar mass, M_w_ = weight average molar mass, N = total number of moles in the sample, N_i_ = number of moles with the molar mass M_i_, m = mass of the whole sample, m_i_ = total mass of all moles with the molar mass M_i_.

### 2.6. Determination of Molar Ratio

For the determination of the BG molar ratio, BG from bread and chyme samples was extracted and precipitated as described in Section 2.6. The so prepared bread and chyme samples, as well as the hBG and cBG extracts, were suspended in 4 mL sodium phosphate buffer (20 mM, pH 6.5) and boiled for 3 min. All prepared samples (extracts, breads and chymes) were combined with 200 µL of lichenase solution (10 U, E-LICHN, Megazyme, Bray, Ireland) and incubated for 1 h at 50 °C under continuous stirring. The incubation was terminated by the addition of 5 mL of sodium acetate buffer (200 mM, pH 4). After removing solids by centrifugation (10 min, 1000× *g*, RT), 5 mL of the supernatant were transferred to a 10 mL volumetric flask. To remove proteins, 50 µL Carrez I (15%, *w*/*v*) and 50 µL Carrez II (23%, *w*/*v*) were added. Sodium phosphate buffer (200 mM, pH 4) was used to adjust the volume. The resulting solution was filtered through a pre-syringe filter (0.45 µm) into a high-performance ion exchange chromatography (HPIC) vial.

For analysis, high-performance anion exchange chromatography HPAEC (Dionex ICS 5000+, Sunnyvale, CA, USA) was used. The system was equipped with a SP single pump (analytical gradient pump), an AS-AP autosampler and a 25 µL sample loop, using the “full loop injection”. An ED electrochemical detector cell with an Ag/AgCl reference electrode and a conventional gold working electrode was used for detection. The gold carbo quad waveform was used. Separation was carried out, on a CarboPac PA 1 (4 × 250 mm) equipped with a CarboPac PA1 guard column (4 × 50 mm), both operated at 25 °C. The mobile phase consisted of (A) 100 mM sodium hydroxide solution and (B) 100 mM sodium hydroxide with 600 mM sodium acetate. Separation of carbohydrates was carried out using the following gradient program: 0–40 min: 95% A, 5% B; 40–55 min: linear increase of B from 5% to 100%; 55–70 min: 95% A, 5% B. Both eluents were degassed with helium and kept under helium atmosphere. The total run time was 70 min at a flow rate of 1 mL/min.

For calibration, analytical standards of 3^1^-β-D-cellobiosyl-glucose (DP3) and 3^1^-β-D-cellotriosyl-glucose (DP4) (both purchased from Megazyme, Bray, Ireland) were used in various dilutions, between 1 and 30 mg/100 mL. All calibrations were found to be linear in the respective calibration range (R^2^ > 0.99). Identity of the peaks, the limit of detection (LOD) and limit of quantification (LOQ) were determined by spiking samples of the cBG extracts, swb and d-swb with the appropriate standard solution prior to extraction. LOD and LOQ were set for a signal to noise ratio of 3 and 10, respectively. Only analyte concentrations above the LOQ were quantified and concentrations below the LOD were expressed as n.d. (not detectable).

### 2.7. Determination of Free Maltose, Glucose and Fructose in Model Breads

The diced model wheat breads were ground to pass a 0.5 mm sieve, using an ultra-centrifuge mill (Retsch, ZM 200, Haan, Germany). Soluble sugars were extracted as described by Schmidt and Sciurba [38]. The analysis was carried out as described in Section 2.7, with the following modified gradient program: 0–40 min: 100% A; 40–55 min: linear increase of B from 0% to 100%; 55–70 min: 100% A. Calibration was done as described in Section 2.7, but using maltose, glucose and fructose in various dilutions between 0.1 and 30 mg/100 mL. Peak identity, LOD and LOQ were determined by addition of the appropriate standard solution to the standard wheat bread prior to extraction.

### 2.8. Viscosity

The dynamic viscosity was determined in the chyme samples obtained from the in vitro digestion of model breads. The rheometer (Haake MARS 60, Thermo Fisher Scientific, Karlsruhe, Germany) was equipped with a circulating thermostat (F32-ME, Julabo GmbH, Seelbach, Germany) and a cone/plate measurement system (diameter = 50 mm, 1° cone angle, measurement gap: 0.052 mm, sample volume: 1 mL). The sample chamber was conditioned to 37 °C by Peltier temperature control modules to simulate human body temperature.

After loading the sample, the measuring system moved to the trim position (measurement gap plus 0.01 mm) and then to the measuring position. The sample was tempered to 37 °C for 120 s at a shear rate ɣ^∴^ of 0.000 1/s. Measurements were performed using CR (controlled rate) rotation and for the shear rate ramp the parameters ɣ^∴^ = 0.001 1/s (Ω = 0.0001677 1/min) to ɣ^∴^ = 100.0 1/s (Ω = 16.77 1/min) were applied with logarithmic data acquisition and continuous display for 100 s. Evaluation of the dynamic viscosity η was carried out using the software Haake RheoWin (version 4.87.0010, Thermo Fisher Scientific, Karlsruhe, Germany).

### 2.9. In Vitro Bile Acid Retention

To determine the bile acid retention capacities of the BG, the total bile acid assay kit (No. STA-631, Cell Biolabs, Inc., San Diego, CA, USA) was used on the chyme samples after in vitro digestion. The preparation of all solutions, standards, as well as the assay were carried out according to the instructions given in the manual. In brief, 2 × 20 µL of appropriately diluted sample or bile acid standard were combined with 150 µL of thio-NAD^+^ solution, using a 96-well microtiter plate, and homogenized. After incubation (5 min, 37 °C), 50 µL of a solution containing 3α-HSD and NADH were added to the standards and one half of the sample wells, while 50 µL of NADH solution were added to the other half of the sample wells. The plate was incubated at 37 °C for 40 min, before the absorbance was read at 405 nm and 630 nm, using a microtiter plate reader (Biotec Synergy, Thermo Fisher Scientific, Schwerte, Germany). All calibrations were found to be linear in the respective calibration range (0–25 µM, R^2^ > 0.99), when plotting the absorbance difference (at 405 nm and 630 nm) against the standard concentration. The available bile acids in the chyme were calculated based on the calibration, according to the manual. The bile acid content of digested swb was used as baseline, to determine the reduction due to the addition of cBG or hBG to the breads.

### 2.10. Statistical Analysis

BG was extracted in triplicate from each flour. The extracts of each flour were combined and homogenized for further analysis. Baking experiments were done once for each recipe. In vitro digestion was done in triplicate for each bread. All analyses were run in triplicate, unless stated otherwise. Statistical analysis was performed using JMP 14.3.0 (SAS Institute, Cary, NC, USA). Data were checked for outliers (Grubb’s test) and evaluation of significant differences was performed using Student’s *t*-Test (for hBG and cBG extracts) or one-way analysis of variances (ANOVA, for breads and digested breads). All differences were considered significant at *p* < 0.05. Where F-values were significant, multiple pairwise comparisons were carried out with the help of Tuckey Post-hoc test to describe the statistical significance between samples.

## 3. Results

### 3.1. Characterization of β-Glucan Extracts from Barley Flours

#### 3.1.1. Compositional Analysis of the β-Glucan Extracts

The approximate composition of the hBG and cBG extracts was determined (Table 1). BG was found the most abundant constituent in both extracts, but with a significantly higher concentration in cBG than in hBG. Also, the raw protein content was substantially higher in cBG compared to hBG. On the other hand, hBG contained significantly higher amounts of total starch, pentosans and ash than cBG. Both extracts had similar moisture contents.

#### 3.1.2. Evaluation of β-Glucan Molecular Structure

**Figure 1:**
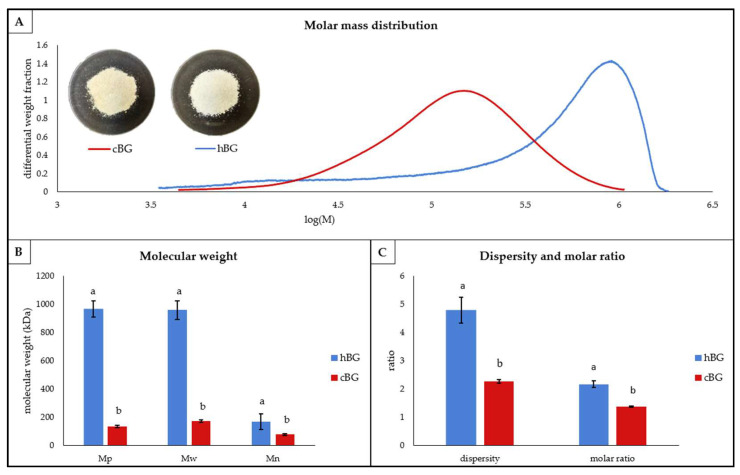
Structural characteristics of high molar mass and control β-glucan (hBG and cBG) presented as: (**A**) molar mass distribution shown as differential weight fraction plotted against the logarithmic molar mass (M), the pictures represent the cBG and hBG extracts; (**B**) peak- (M_p_), weight average- (M_w_) and number average (M_n_) molar mass; (**C**) dispersity and molar ratio. All values are shown as mean ± standard deviation (*n* = 3, *p* < 0.05). Different lower-case letters indicate a significant difference between the extracts for each characteristic.

For further characterization of the hBG and cBG extracts, the structural properties of the extracted BG were investigated (Figure 1).

The differential molar mass distribution of both, hBG and cBG (Figure 1A), was monomodal and approximately similar in shape. For the hBG extract, the peak showed strong fronting. Athough the cBG peak also showed fronting, it was much less pronounced. Furthermore, the BG mass distribution was ranging from log(M) = 3.5–6.3 for the hBG, compared to log(M) = 3.6–6 for cBG. Accordingly, the dispersity (Figure 1C) was significantly lower for cBG. The peak molar mass (M_p_), weight average molar mass (M_w_) and number average molar mass (M_n_) of hBG were significantly larger compared to cBG (Figure 1B). In addition, a bigger discrepancy between M_w_ and M_n_ was evident for hBG compared to cBG. The location of the peak maxima (Figure 1A) further visualized the differences in M_p_ between the two extracts.

The hBG extract also showed a significantly higher molar ratio compared to cBG (Figure 1C). Hence, the relative content of 3^1^-β-D-cellobiosyl-glucose sub-units in the molecule, in relation to the content of 3^1^-β-D-cellotriosyl-glucose sub-units was higher in hBG.

### 3.2. Production and Analysis of β-Glucan Enriched Breads

#### 3.2.1. Bread Quality Characteristics

**Figure 2:**
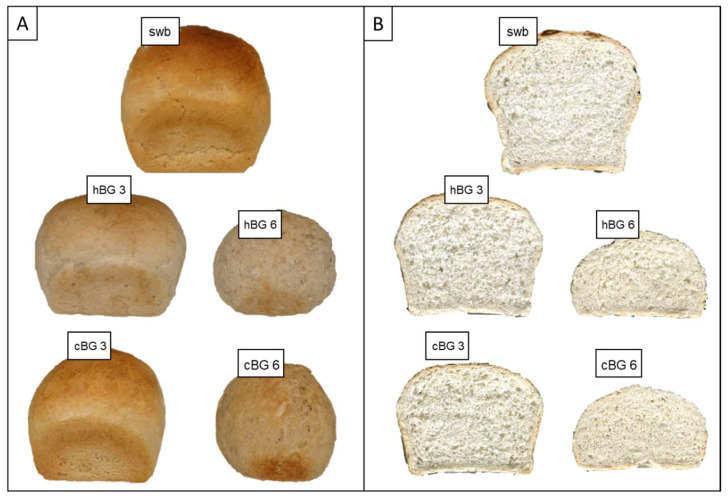
Visual presentation of the standard wheat bread (swb) and breads fortified with 3% of high molar mass β-glucan (BG) or control BG (hBG 3 or cBG 3) and 6% of high molar mass BG or control BG (hBG 6 or cBG 6) presented as: (**A**) whole bread loaves; (**B**) cut-through sections.

Bread loaf and crumb quality characteristics were evaluated visually (Figure 2) and analytically (Table 2).

The swb had a large loaf volume with even and fine crumb structure. While cBG 3 showed a relatively similar color, the other breads were noteworthy paler. It is visible, that with increasing BG content, the bread height decreased, indicating a weaker gluten network and structure. The crumb structure was visibly denser with increasing BG content. There were only minor differences between the hBG and cBG breads with similar BG content, but substantial differences depending on the BG content.

The bake loss was highest for the swb and was found to decrease with increasing BG content. Alongside, the moisture content of the breads increased with increasing BG content. In consequence, the bread weight incresed with increasing BG content in the recipe. The loaf volume was found to decrease with increasing BG content, resulting in highest volume for the swb, followed by hBG 3 and cBG 3 and lowest volume for cBG 6 and hBG 6. At an addition level of 6% BG (based on flour weight) a reduction in volume of more than 50% was found compared to swb. In correlation with an increasing BG content, a decrease in specific loaf volume and an increase in crumb hardness became evident. This resulted in a denser and firmer bread crumb, which is also visible in the cross-section cuts and the pore structure of the hBG 6 and cBG 6 breads. Accordingly, the crumb structure of swb, hBG 3 and cBG 3 breads was rated a six, while the structure of the hBG 6 and cBG 6 was rated an eight (according to Dallmann [35]). The addition of BG, in particular at 6%, also had a negative effect on the bread crumb quality, which became evident by Texture Profile Analysis. The increase of hardness, chewiness and stickiness indicate, that BG fortified breads were more difficult to chew due to a firmer and more resilient crumb that sticks to the teeth. The decrease in elasticity, ballness and cohesion showed a decreased structural integrity upon compression and reduced ability to regain the initial shape after compression. Overall, the largest differences were evident as a result of BG content, but widely independent from the type of BG added.

#### 3.2.2. β-Glucan Content and Free Saccharides

**Figure 3:**
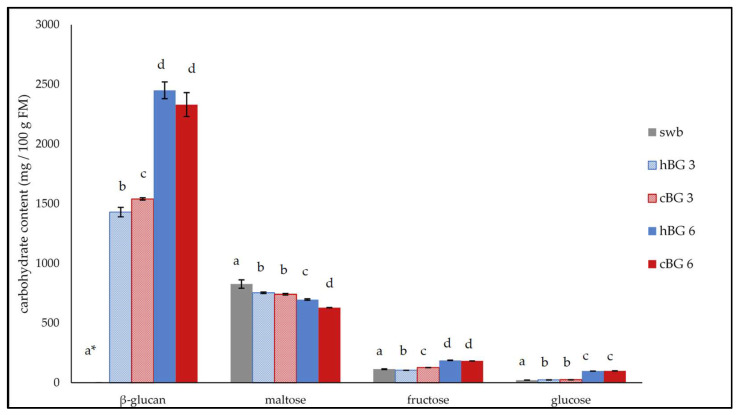
Contents of relevant carbohydrates in standard wheat bread (swb) and in breads fortified with 3% of high molar mass β-glucan (BG) or control BG (hBG 3 or cBG 3) and 6% of high molar mass BG or control BG (hBG 6 or cBG 6 in mg/100 g fresh matter (FM). All values are shown as mean ± standard deviation (*n* = 3, *p* < 0.05). Different lower-case letters indicate a significant difference between the breads regarding each carbohydrate. * BG content was below the limit of quantification of 0.25% and hence, set as “zero”.

The contents of the most relevant carbohydrates were determined in the breads, including BG, maltose, fructose and glucose (Figure 3).

The lowest BG content was determined for the swb and was below the limit of quantification (LOQ) of 0.25%. The BG content in cBG 3 was significantly higher compared to hBG 3, but no significant difference was evident between hBG 6 and cBG 6 breads. The relative BG degradation was higher in the breads with 6% added BG, compared to the breads with 3%. In hBG 3 and cBG 3, 69.9% and 74.8% of the initial BG was detected after baking. Only 61.2% and 58.5% of the initial BG were found in hBG 6 and cBG 6. The BG contents in hBG 6 and cBG 6 were significantly higher compared to hBG 3 and cBG 3.

The maltose content of the swb was significantly higher compared to the breads with added BG. While there was no significant difference regarding the maltose content of the cBG 3 and hBG 3, the hBG 6 contained significantly more maltose than the cBG 6. The fructose content of hBG 6 and cBG 6 was significantly higher compared to the breads containing less BG. The glucose content of the breads was found to increase with increasing BG added to the dough. While the glucose contents of the cBG 3 and hBG 3 breads were significantly higher compared to swb, they were not significantly different from each other. Likewise, the glucose contents of the breads with 6% added BG were substantially higher compared to the breads with less BG added. The contents of the carbohydrates present in the breads were primarily determined by the BG content, rather than the type of BG (hBG or cBG) added.

#### 3.2.3. β-Glucan Structural Characteristics

**Figure 4:**
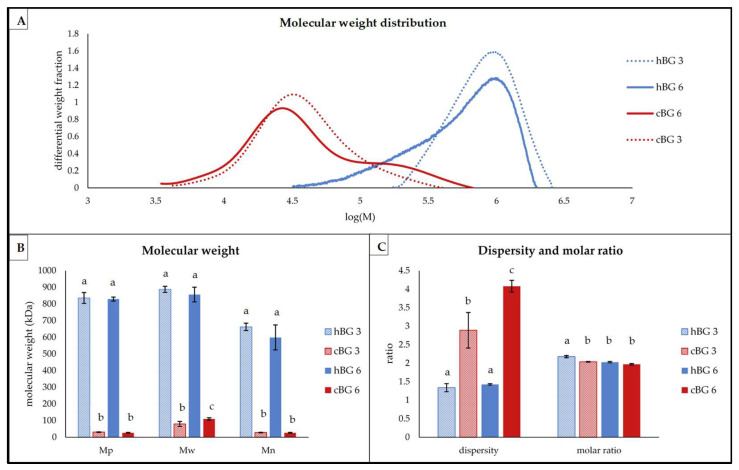
Structural characteristics of β-glucan (BG) present in breads fortified with 3% of high molar mass BG or control BG (hBG 3 or cBG 3) and 6% of high molar mass BG or control BG (hBG 6 or cBG 6) presented as: (**A**) molar mass distribution shown as differential weight fraction plotted against the logarithmic molar mass (M); (**B**) peak- (M_p_), weight average- (M_w_) and number average (M_n_) molar mass; (**C**) dispersity and molar ratio. All values are shown as mean ± standard deviation (*n* = 3, *p* < 0.05). Different lower-case letters indicate a significant difference between the breads for each characteristic.

The BG structural characteristics, including molar mass and molar ratio were determined after bread making (Figure 4). The swb was not included, since the BG content determined in the bread was too low for further characterization.

The hBG had a higher molar mass compared to the cBG (Figure 4A), even after bread making. The peaks of the hBG 3 and 6 were found at approximately log(M) = 6, while the peaks of the cBG were evident at log(M) = 3.2. The cBG peaks were also broader compared to the hBG peaks. The observation was confirmed by the significantly higher dispersity of cBG compared to hBG (Figure 4C). For both cBG peaks, there was strong tailing visible. The cBG 6 even had a shoulder on the right side at log(M) = 5–5.5, which was absent in the cBG 3 peak. In contrast, the hBG peaks showed fronting to various degrees. The hBG 3 showed an approximately symmetric peak with little fronting, while the hBG 6 presented much more pronounced fronting.

The average molar mass values (M_p_, M_w_ and M_n_) were significantly higher for hBG 3 and 6 compared to cBG 3 and 6, but not significantly different from each other (Figure 4B). For cBG 3 and 6, there was a substantially higher M_w_ compared to the M_p_, while M_n_ values were in the same range as the M_p_ values. In contrast, the M_w_ values for hBG 3 and hBG 6 were approximately in the same range as the corresponding M_p_ values. Therefore, the M_n_ of hBG 3 and hBG 6 was substantially lower compared to M_w_ and M_p_. As shown in Figure 4C, the dispersity of the hBG samples was significantly lower compared to the cBG samples, independent from the level of addition. There were no significant differences between the molar ratios of hBG 6, cBG 6 and cBG 3. Only the hBG 3 had a significantly higher molar ratio at 2.18 ± 0.03.

### 3.3. Impact on Functional Properties In Vitro

#### 3.3.1. β-Glucan Structure and Solubility after In Vitro Digestion

**Figure 5:**
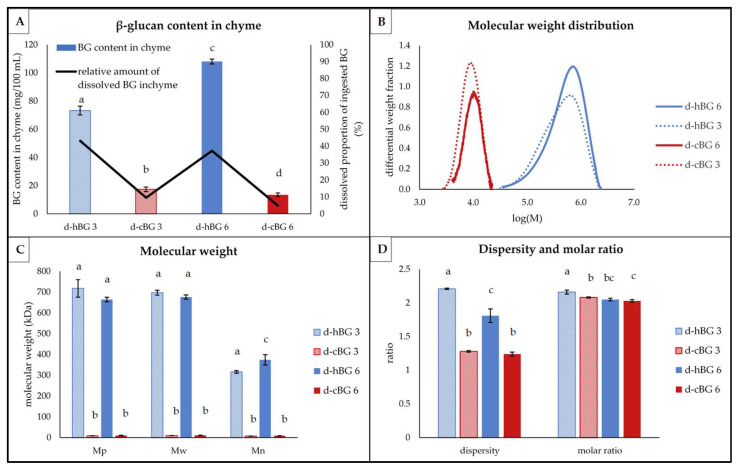
Structural characteristics of β-glucan (BG) present in digested breads breads fortified with 3% of high molar mass BG or control BG (d-hBG 3 or d-cBG 3) and 6% of high molar mass BG or control BG (d-hBG 6 or d-cBG 6) presented as: (**A**) BG content (bars) and proportion ingested BG, which got dissolved in the chyme; (**B**) molar mass distribution shown as differential weight fraction plotted against the logarithmic molar mass (M); (**C**) peak- (M_p_), weight average- (M_w_) and number average (M_n_) molar mass; (**D**) dispersity and molar ratio. All values are shown as mean ± standard deviation (*n* = 3, *p* < 0.05). Different lower-case letters indicate a significant difference between the breads for each characteristic.

To obtain further knowledge on the fate of the two structurally different BG throughout the digestion, the absolute (bars) and relative (line) content and structural properties of the BG dissolved in the chyme after in vitro digestion were determined (Figure 5). Since the BG content of the swb was prior to digestion below the LOQ of 0.25%, it was not characterized further.

The lowest BG contents were found in the d-cBG 6 and d-cBG 3 (Figure 5A). A significantly higher BG content was evident in the d-hBG 3 sample and the highest amount was found in the d-hBG 6. As shown by the black line (Figure 5A), less than 10% of the ingested BG was present in the d-cBG 3 and 6. In contrast, d-hBG 3 and d-hBG 6 contained about 40% of the ingested BG in dissolved form in the chyme.

The BG molar mass distribution was monomodal for all four peaks (Figure 5B). Differences between the peaks were evident based on the type of BG added, not the amount. The d-cBG 3 and 6 showed narrow and approximately symmetric peaks. The peaks of the d-hBG 3 and 6 were visibly broader and showed fronting, similar to the BG in undigested breads. This was supported by the corresponding values for the BG molar mass (M_p_, M_w_ and M_n_) (Figure 5C) and the BG molar ratio and dispersity (Figure 5D).

For d-cBG 3 and 6, no substantial differences regarding the molar mass (M_p_, M_w_ and M_n_) or dispersity could be found, but values were significantly lower compared to the d-hBG 3 and 6 samples. For the d-hBG 3, there was a higher M_n_ than in d-hBG 6, also visible in the more pronounced fronting of the molar mass distribution and significantly higher dispersity. The M_p_ and M_w_ were not significantly different between d-hBG 3 and 6. Between the d-cBG and d-hBG samples there were substantial differences regarding the molar ratio and dispersity. Dispersity in d-hBG 3 was significantly higher compared to d-hBG 6. The highest molar ratio was found in the d-hBG 3 and the lowest in d-cBG 6. There was no significant difference between the molar ratio of d-hBG 6 and d-cBG 6.

#### 3.3.2. Viscosity and Bile Acid Retention

**Figure 6:**
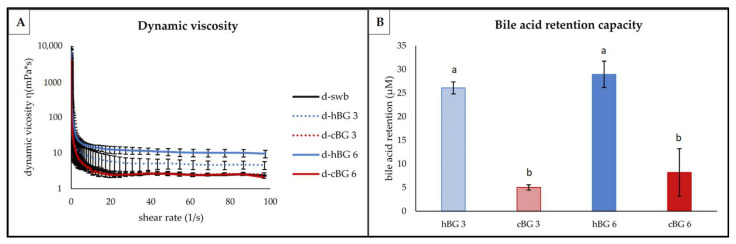
Functional properties of chyme from digested breads fortified with 3% of high molar mass β-glucan (BG) or control BG (d-hBG 3 or d-cBG 3) and 6% of high molar mass BG or control BG (d-hBG 6 or d-cBG 6) presented as: (**A**) dynamic viscosity; (**B**) bile acid retention capacity. All values are shown as mean ± standard deviation (*n* = 3, *p* < 0.05). Different lower-case letters indicate a significant difference between the chyme samples.

Dynamic viscosity and BG bile acid retention capacity were determined from the chyme samples, obtained during in vitro digestion, as an indication of health promoting properties (Figure 6).

No substantial difference in dynamic viscosity (Figure 6A) was evident between the d-swb, d-cBG 3 and d-cBG 6 samples. A noteworthy higher viscosity was apparent for the d-hBG 3 sample at shear rates exceeding 20 1/s. The highest viscosity was determined for d-hBG 6. The addition of a higher amount of hBG in the breads resulted in an overall higher viscosity of the resulting chyme samples. At all shear rates exceeding 20 1/s d-hBG 6 showed substantially higher viscosity compared to all other samples. Determination of the dynamic viscosity showed pseudoplastic (or structurally viscous) flow behavior for all chyme samples.

The results of the bile acid retention of the BG fortified breads were expressed as difference to the bile acid retention of the swb (Figure 6B). There were no significant differences on bile acid retention between hBG 3 and 6 or between cBG 3 and 6, respectively. There was significantly higher bile acid retention by d-hBG 3 and 6 compared to d-cBG 3 and 6, regardless of the level of addition.

## 4. Discussion

The two BG extracts were produced by similar procedures. Hence, all differences in BG structure and functionality between the two extracts originate only from variations in the raw material and are not process induced. The analytical results of the extract compositions are generally in line with previously reported values [26,39,40]. In particular, pentosan and protein contents are in good agreement with the results of Temelli [26]. The author further reported varying BG contents between 69.8% and 89.1% or 86.8–89.1% depending on the pH (between 7 and 9) and temperature (40–55 °C), respectively, during extraction. Although, the optimized extraction conditions were used for the present study, the BG contents are lower than expected, while the starch and pentosan contents exceed the expectations [26,40]. The reason behind could be in the temperature and pH distribution in the extraction slurries, despite the constant stirring. As a result, areas closer to the heating surface may have exceeded 60 °C, which was previously stated to increase starch contamination due to gelatinization [39]. This effect was, due to the observed higher viscosity, noteworthy stronger for the hBG extract, explaining the lower BG content. The molar mass of hBG was higher compared to both, cBG and literature reports for optimized extraction conditions [26,39]. Molar mass is considered a key factor for high viscosity of BG solutions [7]. The BG extraction yield is further affected by the BG solubility, as well as the covalent and non-covalent association with cell-wall components. Differences in extraction yield of up to 5% between barley varieties have been reported previously [39]. Due to the higher molar ratio, the hBG is expected to have a lower solubility compared to the cBG [41]. The reason behind is the more linear structure due to the higher proportion of DP3 sub-units in the molecule. This allows for tighter packing of the BG chains, leaving less space for water molecules to interact in between. For further purification of the extracts, the use of endogenous enzymes would be required [7].

The BG-dependent bread quality deterioration is consistent with previous studies [42,43,44]. The reason behind is the high gelation potential of BG, resulting in increased water binding and viscosity of the dough. Based on the structural differences between hBG and cBG, one could expect a less severe impact of the cBG, since dough viscosity and water binding would be affected less by the lower molar mass BG [44]. This could not be confirmed. It is possible, that the structural differences between hBG and cBG were not substantial enough to result in noteworthy different bread quality at the respective BG contents. The water binding capacity of the BG is also evident in the higher moisture contents of BG fortified breads compared to the swb. As there is less free water available in the dough, the gluten network development is hindered. As a result, the gas holding capacity of the dough is reduced, leading to the smaller loaf volumes [42,43,44]. Based on the existing literature, one would further expect a relation between the BG molar mass and the resulting loaf volume. Previous studies have reported a relation between high viscosity and smaller loaf volumes [42,43]. A corresponding difference could only be observed between hBG 6 and cBG 6, but not between hBG 3 and cBG 3. The reduced bake loss with increasing BG content confirms the findings of Cleary, Andersson and Brennan [43]. The results further suggest, that the BG content has a greater impact on the crumb texture of the resulting breads compared to BG molar mass. The influence of BG content on the crumb texture has already been demonstrated previously [43,45]. Ortiz de Erive et al. [46] posited two possibilities that would explain the decrease in elasticity corresponding to the increase in BG content in bread. The first possibility is that the increase in BG content leads to dilution and partial dehydration of gluten, which in turn leads to a decrease in gas-holding capacity and strength of the gluten network. The second possibility is that insufficient or incomplete starch swelling and gelatinization occurs during baking, preventing the formation of an interconnected sponge-like structure. A possible solution to obtain high quality BG enriched breads could be in the adjustment of the water content. Adding more water can increase the elasticity values of wheat breads due to the improvement of starch swelling [46]. Similar conclusions can be drawn for the cohesion. Thus, it can be concluded that the addition of BG substantially impacts the various bread quality parameter, compared to lower addition levels. However, the quality deterioration was based on the level of BG addition, while molar mass and structure generally had no impact on the bread quality.

The decrease in maltose due to the increasing BG content may be explained by the limited starch access of the amylases, due to high viscosity and water binding of the BG. Since less maltose is released, the yeast is obtaining glucose from the BG and sucrose present. The results further show a substantial discrepancy between the amount of BG added to the dough and the BG found after baking. According to the existing literature, this is due to the enzymatic hydrolysis of BG by β-glucanase, present in the flour and released by the yeast [20,22]. This is also in line with the increase in free glucose as a result of higher BG addition level. On the other hand, there is evidence, that BG with higher molar ratio has better resistance against enzymatic hydrolysis [23], which cannot be confirmed by the BG contents of the hBG and cBG breads. Despite the initially higher molar ratio, hBG 3 even has a lower BG content compared to cBG 3. However, BG hydrolysis leaving fragments of DP3 or bigger would not be detected by the photometric determination. Considering the requirement of 1.0 g of oat or barley BG per serving (resulting in a total consumption of 3 g/day) [13], the cBG 6 and hBG 6 breads would warrant the health claim for a reduction of blood cholesterol [47]. The swb, cBG 3 and hBG 3 breads do not contain sufficient amounts of BG.

Regarding the structural properties of BG, the application during bread making process had different impact on hBG and cBG. For cBG 3 and 6, the molar mass distribution among the BG population became more inhomogeneous, visible by the broader peak shape and increased dispersity, compared to the cBG extract. In contrast, the dispersity of hBG decreased noteworthy from 4.8 in the extract (Figure 1) to approximately 1.4 in the final breads (Figure 4). Likewise, the BG molar mass reduction during the bread making is, with approximately 50%, noteworthy higher for cBG compared to the 10% reduction for hBG (based on the M_w_-value), which is a moderate reduction compared to literature reports [48]. Opposing results of molar mass reduction for high molar mass BG during bread making, but not for low molar mass BG were reported by Cleary, Andersson and Brennan [43]. This suggests, that a higher molar ratio improves the resistance to β-glucanase, independent from the molar mass [23]. For hBG 3 and 6, the dispersity decreased and the M_n_ increased compared to the hBG extract. This indicates that primarily the low molar mass BG populations were degraded during bread making. In contrast, the BG hydrolysis in cBG is more evenly distributed between molecules of various masses, resulting in a shift towards lower masses. Also, M_n_, M_w_, and M_p_ were reduced evenly in cBG. Another factor for the high molar mass reduction could be the lower viscosity of the dissolved BG, allowing for better accessibility of the BG for enzymatic hydrolysis [20]. A heat induced depolymerization of BG is unlikely to be a considerable factor [22]. Another point of interest are the changes in BG molar ratio during bread making, with added hBG and cBG. While the molar ratio of hBG remained unaffected, the molar ratio of the cBG increased significantly. This indicates a preferred hydrolysis of DP4 sub-units in cBG 3 and cBG 6. Considering the BG content and molar mass, the cBG got degraded more rapidly in the dough, but was still detected as BG (i.e., DP3). In the final breads, the molar ratio of hBG and cBG were nearly similar at approximately 2. Overall, the structural differences between the BG extracts showed no impact on bread quality and the content of BG and other relevant carbohydrates. Differences between the breads are based on the BG content. Only regarding molar mass distribution and molar ratio, the bread making showed different impact on hBG and cBG.

The BG structural differences show an impact on the solubility during in vitro digestion. A possible reason behind can be the larger molar mass of hBG, which has been reported for better solubility compared to lower molar mass BG [11,17,49]. From the low BG contents of the d-cBG 3 and 6, it can be assumed, that a large fraction of the BG remained undissolved and without health promoting activity in the pellet. It was reported previously, that after in vitro digestion of breads containing oat BG the level of dissolved BG was ranging between 36% and 54% [50]. While the BG content of d-hBG 3 and 6 is within this range, d-cBG shows a noteworthy lower solubilized proportion. It also should be noted that the viscosity is limited by the solubility of BG [17,51]. The relation between BG content and chyme viscosity can be described as follows. Upon reaching a critical BG concentration the polysaccharide chains overlap, causing viscosity to increase exponentially with BG content and molar mass. This is due to molecular enlargement due to specific molecular associations or the presence of aggregated particles [52,53,54]. In this context, viscosity is limited by the solubility of BG [17,51]. As a result, the viscosity of d-cBG 3 and 6 is lower compared to hBG 3 and 6 and the entrapment of the bile acids is compromised [17,20,41,52].

The results further show a molar mass reduction from the fresh bread to the in vitro digested breads, which is only partially supported by the existing literature. While Rieder et al. [55] reported a decrease in molar mass, others found no changes in BG molar mass during in vitro digestion [43]. Hence, a general conclusion regarding the maintenance of BG molar mass during digestion is not possible. Otherwise, a relation to the molecular properties, such as the initial molar mass is possible. To this end, high molar mass BG was reported to decrease significantly in weight from about 1300 kDa to 700 kDa, while lower molar mass BG (approximately 285 kDa) tolerated the in vitro digestion better [48,56]. In the present study, cBG and hBG were both found at a substantially reduced molar mass after digestion. The percentage reduction of the cBG molar mass during digestion was ever higher compared to high molar mass hBG. Since the molar ratio of cBG and hBG was very similar in the breads, this can be excluded as factor for the differences in molar mass reduction. There were overall only minor changes in BG molar ratio during digestion. This could indicate an approximation for an ideal molar ratio around 2, with maximum resistance to enzymatic hydrolysis, which has not been reported before. For cBG, it can further be seen, that the remaining larger molar mass populations in the bread were hydrolyzed or not soluble during the digestion, leaving only fragments with approximately 10 kDa detectable in d-cBG 3 and 6. This is not evident in the comparison of the hBG and d-hBG samples.

The most common explanation behind the LDL-cholesterol lowering properties of BG is based on the formation of a highly viscous gel in the intestine. This is disrupting the interaction of the micelles with the luminal membrane transporters on the intestinal epithelium, preventing the absorption of free bile acids from the chyme [15,57]. As a consequence, the bodies de novo synthesis of bile acids is triggered, requiring cholesterol as precursor. Hence, the dynamic viscosity after digestion and the bile acid retention capacity can be used to estimate the BG health promoting properties. The higher viscosity of the d-hBG samples compared to the other three samples is consistent with the higher BG content and molar mass of hBG and in line with existing literature [17]. A visibly higher viscosity was observed already during extraction from barley flour and to some degree maintained even throughout the digestion. Due to the low BG content and molar mass of the d-cBG samples, very low bile acid retention capacity was evident. This indicates lower health promoting properties of cBG 3 and 6, compared hBG 3 and 6, even when breads contained similar BG contents. Moreover, with lower BG content the hBG 3 showed even better bile acid retention compared to cBG 6. Despite reports on bile acid retention by low viscosity BG [58], the molar mass and in turn viscosity were identified as crucial factors in this study. This is in line with the reports by Makela et al. [59].

Overall, the suitability for food application and health promoting properties was found to be strongly dependent on the molecular properties of the applied BG. Despite using the same BG concentrations, and the similar fate on BG content, structural differences were magnified during baking. During the subsequent in vitro digestion the differences between hBG and cBG became clearly evident. A correlation between BG content and molecular properties in the raw material and total bile acid retention during digestion should further be substantiated, using more complex digestion models or human dietary studies.

## 5. Conclusions

In this study, two structurally different BG populations (hBG and cBG) were investigated as health promoting ingredient in bread making and subsequent in vitro digestion. Despite similar BG content reduction, the impact of bread making on structural properties was different for hBG and cBG. Due to the molecular differences, magnified by bread making, solubility, viscosity and bile acid binding during the in vitro digestion process were affected. The results indicated better health promoting properties for hBG compared to cBG.

To date, this is the first study demonstrating the fate of two structurally different barley BG from native BG-extracts, over the utilization as bread ingredient until the digested food product. There is evidence for better cholesterol lowering properties of the higher molar mass BG. Moreover, even at similar BG concentrations in the model breads, the structural properties were confirmed as key factor for the fate and functionality during in vitro digestion. These results provide further insight into the relevance of BG structural properties, to obtain a product with the best health-benefits possible. Further investigations are necessary to understand the ideal combination of BG molecular properties, also considering various food products. In addition, human dietary studies are required to further substantiate the results of this study.

## Figures and Tables

**Table 1:** Approximate composition of high molar mass (hBG) and control (cBG) β-glucan extracts from two commercial barley flours ^#^.

Component	Content in hBG Extract (%)	Content in cBG Extract (%)
moisture	9.6 ± 0.1 ^a^	9.5 ± 0.1 ^a^
β-glucan	63.3 ± 2.1 ^a^	69.9 ± 0.9 ^b^
starch	13.2 ± 0.8 ^a^	5.6 ± 0.3 ^b^
protein	4.1 ± 0.1 ^a^	10.2 ± 0.1 ^b^
ash	2.21 ± 0.01 ^a^	0.58 ± 0.02 ^b^
pentosans	1.09 ± 0.07 ^a^	0.04 ± 0.01 ^b^

^#^ Results are shown as mean ± standard deviation. Values in one row followed by the same superscript lower-case letter are not significantly different (*p* < 0.05).

**Table 2:** Bread and crumb quality parameter for standard wheat bread (swb) and breads fortified with 3% of high molar mass β-glucan (BG) or control BG (hBG 3 or cBG 3) and 6% of high molar mass BG or control BG (hBG 6 or cBG 6). Each recipe was baked once.

	Sample	swb	hBG 3	cBG 3	hBG 6	cBG 6
	total dough weight (g)	167	167	168	167	167
	total bread weight (g)	139	145	145	149	150
	bake loss (%)	16.8	13.2	13.7	10.8	10.2
	loaf moisture content (%)	39.4	41.3	44.1	44.6	43.8
	loaf volume (cm^3^)	510	410	400	190	220
	crumb structure category (according to [35])	6	6	6	8	8
**Texture Profile Analysis**	hardness (N)	6.7	9.4	9.7	35.1	41.8
	elasticity (%)	98.0	98.1	95.8	88.9	94.1
	cohesion	0.73	0.74	0.76	0.51	0.51
	ballness	0.40	0.39	0.42	0.19	0.19
	stickiness (g∙s)	−1.2	−0.7	−0.4	−258	−207
	chewiness (N)	4.78	6.80	6.35	15.78	19.95

## Data Availability

Data is contained within the article.
